# Omega-3 Eicosapentaenoic Acid (EPA) Rich Extract from the Microalga *Nannochloropsis* Decreases Cholesterol in Healthy Individuals: A Double-Blind, Randomized, Placebo-Controlled, Three-Month Supplementation Study

**DOI:** 10.3390/nu12061869

**Published:** 2020-06-23

**Authors:** Amanda Rao, David Briskey, Jakob O Nalley, Eneko Ganuza

**Affiliations:** 1 RDC Clinical, Brisbane 4006, Australia; amanda@rdcglobal.com.au (A.R.); d.briskey@uq.edu.au (D.B.); 2 School of Medicine, University of Sydney, Sydney, NSW 2006, Australia; 3 School of Human Movement and Nutrition Sciences, The University of Queensland, Brisbane, QLD 4067, Australia; 4 Qualitas Health, Houston, TX 77056, USA; jnalley@qualitas-health.com

**Keywords:** long-chain omega-3 polyunsaturated fatty acids, eicosapentaenoic acid, polar lipids, galactolipids, cardiovascular health, cholesterol, very-low-density lipoprotein, dietary supplements, microalgae, *Nannochloropsis*

## Abstract

The aim of this trial is to assess the effect of Almega^®^PL on improving the Omega-3 Index, cardio-metabolic parameters, and other biomarkers in generally healthy individuals. The benefits of long-chain omega-3 fatty acids for cardiovascular health are primarily built upon mixtures of docosahexaenoic (DHA) and eicosapentaenoic acids (EPA). Highly purified EPA therapy has proven to be particularly effective in the treatment of cardiovascular disease, but less is known about the benefits of EPA-only supplementation for the general healthy population. Almega^®^PL is a polar rich oil (>15%) derived from the microalga *Nannochloropsis* that contains EPA (>25%) with no DHA. Participants (*n* = 120) were given a capsule of 1 g/day of either Almega^®^PL or placebo for 12 weeks. Differences in the Omega-3 Index, cardiometabolic markers, and other general health indicators were measured at the baseline, six, and 12 weeks. Compared to the placebo group, Almega^®^PL supplementation significantly increased the Omega-3 Index and EPA concentration from 4.96 ± 0.90 and 0.82 ± 0.37% at the baseline to 5.75 ± 0.90 and 1.27 ± 0.36 at week 12, respectively. Very-low-density lipoprotein cholesterol (VLDL) decreased by 25%, which resulted in a significant decrease in total cholesterol compared to the placebo. Interestingly, the decrease in VLDL was not associated with an increase in LDL, which seems to be a benefit associated with EPA-only based formulations. Collectively, these results show that Almega^®^PL provides a natural EPA-only option to increase EPA and manage cholesterol levels in the general population.

## 1. Introduction

According to the World Health Organization, cardiovascular diseases (CVD) are the leading cause of death globally [1]. In the USA, an estimated 14.7% of the cardiometabolic deaths (54,626) each year are linked to low intake of the long-chain omega-3 polyunsaturated fatty acids (LCn-3 PUFA), eicosapentaenoic (EPA) and docosahexaenoic (DHA) [2]. Seafood consumption is low in the Western diet, 95% of the USA population does not consume enough EPA and DHA [3]. Therefore, health authorities in the USA [4], Australia [5], and other Western countries with similar dietary habits recommend the daily intake of 160–610 mg of LCn-3 PUFA.

Cardiovascular health benefits of DHA and EPA supplementation are now supported by a large body of evidence, including the decrease of cardiometabolic risk factors such as triglycerides [6], cholesterol [7], blood pressure [8], and subsequently death [9]. The decrease in atherogenic lipid levels by LCn-3 PUFA is driven by the decrease in the very low-density lipoprotein cholesterol (VLDL) secretion by the liver, which typically coincides with a decrease in the triglycerides that are transported by this lipoprotein [10]. The mechanistic explanation as to how LCn-3 PUFA effect VLDL production is unknown but could involve a change in liver function associated with an increase in whole-body fatty acid oxidation that ultimately results in reduced liver fat. Whether the decrease in VLDL can drive a decrease in total cholesterol (TC) is controversial [6], but recent reports indicate that it might depend on the type of LCn-3 PUFA used [7,11]. DHA-containing formulations seem to increase low-density lipoprotein cholesterol (LDL) in response to the VLDL decrease, while formulations containing EPA-only are able to decrease VLDL without increasing the LDL [7,11]. Therefore, an EPA-only formulation could potentially impact both cholesterol and triglyceride levels.

The body of evidence for LCn-3 PUFA health benefits is largely built on fish sources and very little is known about the structural and compositional impact of plant-based sources. Traditionally LCn-3 PUFA supplements are produced using oil extracted from wild caught fish. However, fish do not synthetize enough LCn-3 PUFA. Instead, they obtain it from phytoplanktonic microalgae throughout the food chain. Through this process, LCn-3 PUFA are bioaccumulated in complex mixtures of DHA and EPA that are predominantly esterified into neutral lipids. Instead, the primary microalgal producers often synthetize unmixed LC-PUFA that are conjugated to polar lipids. For instance, the microalga *Nannochloropsis* produces EPA without the DHA, which is typically present in other LCn-3 PUFA natural sources. This is important because EPA might have different benefits in cardiovascular health than DHA [9,10], but most supplementation studies conducted are based on supplements with DHA + EPA mixtures.

Plant-based Almega^®^PL derived from *Nannochloropsis* is the first photosynthetic source of LCn-3 PUFA available for human consumption in the USA. Besides offering a natural source of EPA-only (25%), it contains polar lipids (15%) rich in galactolipids and phospholipids, which provides nutritional characteristics that are different from other forms of LC-n3 PUFAs. This unique polar lipid composition confers surfactant properties that trigger the formation of micelles in the stomach. Those spontaneous emulsions facilitate the digestion and the delivery of LCn-3 PUFA while minimizing fishy burps and aftertaste. Kagan et al. [12] demonstrated that LCn-3 PUFAs in Almega^®^PL was more bioavailable than other forms of LCn-3 PUFA.

This novel form of LCn-3 PUFA opens the opportunity to investigate the health benefits of other forms of LCn-3 PUFA that are relatively unexplored. Besides the absolute values of LCn-3 PUFA intake, both the composition (EPA only) as well as the structure (polar rich) of this plant-based source may lead to a better understanding of the cardioprotective and health benefits of LCn-3 PUFA. The aim of this study is to assess the effectiveness of Almega^®^PL on improving the Omega-3 Index (O3I), cardio-metabolic parameters, and health-related quality of life in generally healthy men and women.

## 2. Material and Methods

### 2.1. Clinical Trial Design, Registration and Ethical Approval

This trial was conducted in compliance with the current International Conference on Harmonization (ICH) Guideline for Good Clinical Practice (GCP), the Therapeutic Goods Administration (TGA), the Notice for Guidance on Good Clinical Practice, and the ethical guidelines outlined in Additional Ethical Considerations. The trial was approved by the Bellberry Limited Human Research and Ethics committee (approval number 201712916) and registered on the Australia New Zealand Clinical Trials Registry (ACTRN12618000275268).

This study was a double-blind, parallel design, randomized clinical trial with a 3-month supplementation period utilizing an active and a placebo arm, assessing the effect of Almega^®^PL on cardio-metabolic parameters and inflammatory markers in men and women with a low O3I. This study was conducted between June 2018 and January 2020 in Brisbane, Australia.

### 2.2. Participants

Potential participants were recruited from databases and public media outlets. Following preliminary screening via telephone, potential participants attended the clinic for an information session and provided their consent for inclusion in the trial. Consenting participants underwent a health assessment including lifestyle, blood pressure, current medication, and medication history (i.e., history of disease and illness), the data of which was used for comprehensive screening and contextual data for the study.

A total of 120 otherwise healthy volunteers (males and females) over 25 years of age were recruited from Brisbane and surrounding areas to take part in this study. Participants were excluded if they had an unstable or serious illness (including but not limited to kidney, liver and gastrointestinal disease, any heart conditions or diabetes), had total cholesterol levels for which drug therapy is indicated (>9 mmol/L) [13] and were subsequently referred to a general practitioner, were on cholesterol-lowering medication, had malignancy or a treatment for malignancy within the previous 2 years, were receiving or prescribed coumadin (Warfarin), heparin, dalteparin, enoxaparin or other anticoagulation therapy, were active smokers, had chronic past and/or current alcohol use (>14 alcoholic drinks per week), had serious mood disorders (such as depression or bipolar disorder) or any other conditions in which the opinion of the investigators made the participant unsuitable for inclusion. Participants were also excluded if they were found to have any allergic reactions to any of the ingredients in the active or placebo formula or had participated in any other clinical trial during the past month. Pregnant or lactating women were excluded from the study.

### 2.3. Investigational Products

The investigational product, supplied by Qualitas Health (Houston, TX, USA) under the brand name iWi, was a vegetarian capsule-form containing 1 g Almega^®^PL, an ethanol extract from whole-cell *Nannochloropsis* sp. microalga rich in galactolipids and phospholipids. Each capsule was standardized to contain 250 mg EPA, 150 mg of polar lipids, as well as 40 mg of arachidonic acid (ARA; 20:45 n-6) and 90 mg of palmitoleic acid (16:1 n-7). This ingredient also contained 23 mg of phytosterols and 150 mg of chlorophyll, 764 µg lutein, 387 µg zeaxanthin, and 541 µg beta-carotene analyzed according to Eurofins methods (Des Moines, IA, USA). The placebo contained 1 g of soy oil housed in an opaque gel capsule to appear identical to the test product. Soybean oil was selected as a placebo because it does not contain any of the active constituents described above. The placebo and active product were manufactured by Qualitas Health under batch number 170731AP01and 180104AP01. The product is registered for use as a new dietary ingredient (NDIN) in the USA.

### 2.4. Intervention and Study Procedures

Once enrolled in the study, participants were randomly allocated to either the active intervention group (*n* = 60 per group) or placebo comparator group (*n* = 60 per group). At enrollment, participants provided a baseline blood sample (approximately 20 mL) for analysis of pre-treatment blood markers and measures were taken for: body composition, dietary intake (diet diary), mood, sleep quality, quality of life (QOL) and fatigue. Their O3I and fatty acid profile were measured via a finger prick test (OmegaQuant^TM^, South Falls, SD, USA) [14]. Once all baseline measures were taken, participants were instructed to take one capsule of the active or placebo product per day, taken orally with water at breakfast. This regime was selected on the basis of current standard dosing guidelines for the investigational product. Participants were then required to attend the study site after six (mid-point) and 12 (completion) weeks of supplementation to repeat baseline measures.

Participants were asked to maintain their usual level of physical activity and diet for the duration of the study. This was assessed by questionnaire and a dietary intake diary. Any changes to the usual activity level and/or diet were considered when assessing results. Participants were also monitored for any potential adverse reactions and compliance with the protocol. The compliance success rate was evaluated by counting the allocated product remaining in the container at the end of the study. Participants that either did not return the bottles or returned bottles containing more than 5% of the assigned capsules were considered non-compliant.

### 2.5. Randomisation and Blinding

Randomization of the products was conducted independently of the investigators using Random Allocation Software V2018, Sealed Envelope, London, UK (www.sealedenvelope.com). Neither the participants nor the researchers knew which product was allocated to each subject until completion of all statistical analyses.

### 2.6. Outcome Measurements

O3I (primary outcome) measures EPA and DHA in red blood cell membranes as % of total fatty acid, an indicator of the omega-3 nutritional status. O3I, arachidonic acid (ARA), EPA, docosapentaenoic acid (DPA; 22:5 n-3) and DHA in erythrocytes were measured using the finger prick test [14]. Blood pressure, fasting total cholesterol (TC), triglycerides, low-density lipoprotein cholesterol (LDL), and high-density lipoprotein cholesterol (HDL) were analyzed in duplicate using kits and calibrators for a clinical chemistry analyzer (BK400, Biobase, Jinan, China). VLDL (=TC-HDL-LDL), non-high-density lipoprotein cholesterol (non-HDL-C) (=TL-HDL), and TC/HDL ratio were all calculated. Anthropometric measures included height, weight, body mass index (BMI), and waist and hip circumference. Safety Markers and Adverse Reaction measurements included alanine transaminase (AST), aspartate transaminase (ALT), gamma-glutamyl transferase (GGT), bilirubin, creatine, and glucose (BK400, Biobase, Jinan, China). Adverse events and changes in medication use, either reported by the patient or noticed by a Medical Supervisor, were recorded throughout the study. Oxidative stress markers analyzed included reactive oxygen metabolites (d-ROMS) and homocysteine (HOM). Inflammatory status was assessed with C-reactive protein (hs-CRP; BK400, Biobase, Jinan, China), tumor necrosis factor alpha (TNF-α), and interleukin-6 (IL-6) TNF- α and IL-6 were analyzed using Milliplex high sensitivity T cell panel from Merck (Macquarie Park, New South Wales, Australia) and analyzed on the Luminex 200 (Luminex Corporation, Austin, Texas, USA).

Questionnaires were used to assess mood (The Profile of Mood States Questionnaire (POMS)) [15] and general quality of life that are potentially associated with high inflammation [16] and may be affected by LCn-3 PUFA supplementation. The Pittsburgh Sleep Quality Index (PSQI) was used to measure sleep quality and pattern [17], the Multidimensional Symptoms Fatigue Inventory (MSFI) for fatigue [18], The Short Form Health Survey questionnaire (SF-36) for health-related quality of life (HRQoL) (including pain) [19], The Food Frequency Questionnaire (FFQ) and the 24 h Dietary Recall (24HR) used at baseline and week 12 to obtain detailed information about calories consumed [20]. The questionnaire specifies the frequency of consumption and portion size of foods with a high content of LCn-3 PUFA such as salmon, tuna, or shellfish.

### 2.7. Statistical Analyses

Power and sample size calculation was performed on the primary outcome (O3I) using G*Power 3.0 v3.1.9.3 (Department of Psychology, University of Düsseldorf, Germany). Sample size calculation was based on a student t-test (two independent samples), assuming 20% difference between the group’s deltas (endpoint minus baseline), reflecting a medium effect size of 0.5. The 20% difference was considered potentially relevant based on previous O3I response reports for baseline O3I levels typically associated to Western diets [21,22]. A total of 120 participants (60 per group) were recruited (accounting for 20% dropouts) to secure 80% power to detect such change at the two-sided significance at 5% level. Results for the per protocol (PP) population were analyzed with R [23] using a range of native statistical functions, and in some cases, functions from the packages *tidyverse*, *dplyr* and *ggplot*. All data was first tested for normality before any other test was conducted and baseline data was compared for differences between groups. The different outcomes were then analyzed as a comparison of the change from baseline (Δ values) between groups (active vs. placebo). Based on the distribution of data, Welch two-sample unequal variance *t*-tests and Wilcoxon Ranks sum tests were used to compare two-tailed differences between groups. Differences were considered statistically significant at a *p* value < 0.05. Intervention effect was also tested using an analysis of covariance model (ANCOVA) adjusting for baseline parameters. Additional *post hoc* analyses were carried out on a subgroup of participants (*n* = 76) with a baseline cholesterol level between 5.5–9 mmol/L which was considered to be outside the upper reference range and therefore at risk, but not medicated [13].

## 3. Results

### 3.1. Participants

**Figure 1:**
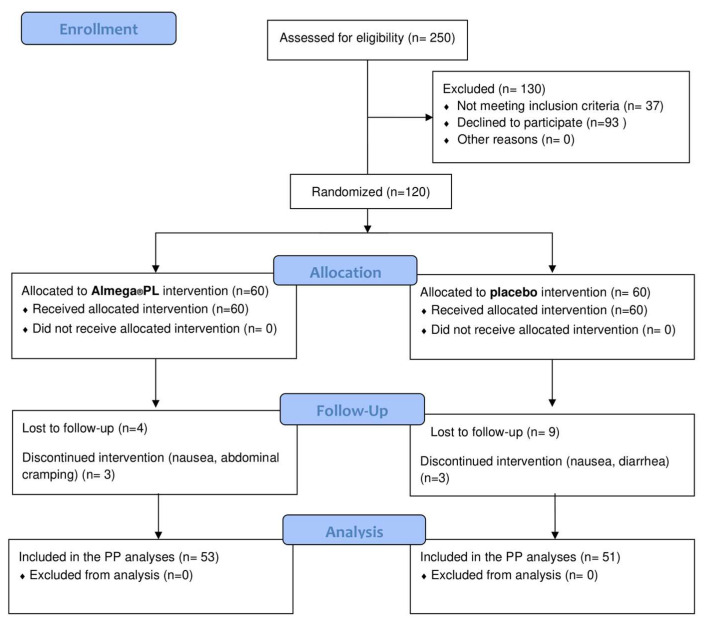
A total of 120 participants were randomized to receive Almega^®^PL or placebo treatment. Study completers (*n* = 104) included participants who completed the week 12 visit. Participants who dropped out were not included in the statistical analysis.

Of the 120 randomized participants, 104 completed the study (53 active and 51 placebo), illustrating the good tolerance to the intervention product (Figure 1). There were seven withdrawals in the active treatment group, three of which were due to adverse events (nausea and abdominal cramping). There were nine withdrawals in the placebo treatment group, three of which were due to adverse events (nausea, diarrhea). Other withdrawals were unrelated to the product.

The compliance success rate in the active (92.5%) and placebo (92.5%) group was not statistically different. There was no change or difference between groups in exercise or dietary intake during the study.

Both groups were well matched, with no statistical differences at baseline for all measures (Table 1), with a similar distribution of males and females in each treatment group.

### 3.2. Omega-3 Status

**Figure 2:**
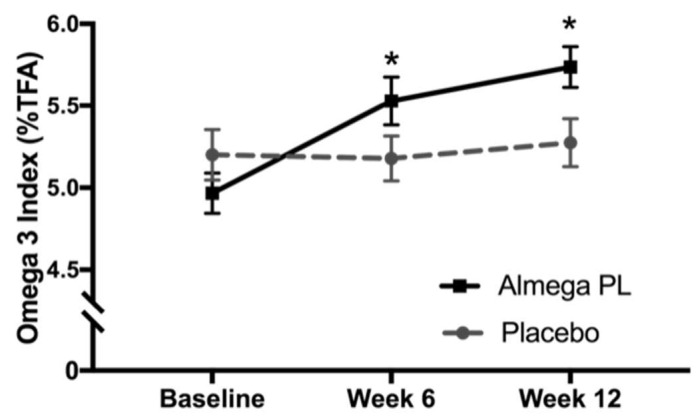
Omega-3 Index, or EPA + DHA % of total erythrocyte fatty acids, measured at baseline, week 6, and week 12. Values represented as mean ± SD, * change from baseline (Δ values) significantly different between groups, *p* < 0.05.

Baseline O3I was not statistically different between the active and placebo groups at the baseline (4.96 ± 0.90 and 5.22 ± 1.08% respectively). The change in O3I at week 6 and week 12 was significantly higher in the Almega^®^PL group compared to placebo (*p* < 0.001) (Figure 2). At week 6 the O3I was 5.51 ± 1.05% and further increased to 5.75 ± 0.90% at week 12. The baseline erythrocyte concentrations of ARA, EPA and DHA were not different between groups at baseline, however, EPA and DPA significantly increased in the Almega^®^PL group at week 6 and week 12 compared to the placebo group. (Table 2). DHA and ARA in red blood cells did not change in either treatment group (Table 2).

### 3.3. Cardiometabolic Markers

Almega^®^PL supplementation significantly decreased total cholesterol (TC) and VLDL cholesterol when compared against placebo in the PP population (n = 104) on week 12 (Table 3). TC decreased by 3% and VLDL decreased by 25% with respect to baseline. No statistical differences were observed in TC, HDL, LDL, triglycerides, TC:HLD ratio, and non-HDL-cholesterol for the PP population.

**Figure 3:**
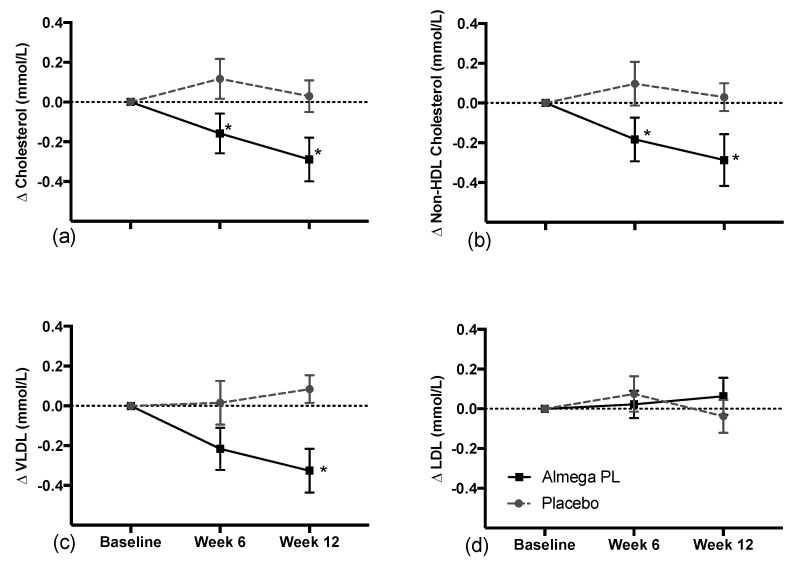
Change in total cholesterol (**a**), non-HDL-cholesterol (**b**), VLDL (**c**) and LDL (**d**) in plasma (mmol/L) of 76 participants with higher baseline cholesterol over the 12-week intervention Values represented as mean ± SEM, * change from baseline significantly different to placebo, *p* < 0.05.

Cardiometabolic parameters were further analyzed for a subgroup (n = 76) of participants with higher (>5.5 mmol/L) baseline TC levels (Table 3, Figure 3). The decrease in TC (4%) and VLDL cholesterol (27%) on week 12 with respect to baseline was more pronounced in the subgroup. The subgroup analyses showed that the TC delta values were significantly different starting from week 6, and that difference was even higher on week 12. In addition, the subgroup showed differences in the deltas of non-HDL-cholesterol for both weeks 6 and 12. The HDL and LDL did not change significantly between groups over the intervention period.

### 3.4. Antropometric Measures

There were no differences between groups for any anthropometry measure at the baseline. Supplementation with Almega^®^PL for 12 weeks resulted in a significant reduction in hip circumference and body weight (adjusted for age and baseline BMI) compared to the placebo group. Hip circumference significantly increased from the baseline at week 12 in the placebo group (Table 4).

### 3.5. Safety Markers and Adverse Reactions

All safety markers of liver toxicity were within the normal range at the baseline and at week 12 for both treatment groups, indicating that the Almega^®^PL is well tolerated and has no safety concerns. Following 12 weeks of supplementation, the Almega^®^PL group had a reduction in AST concentration (*p* = 0.0127) compared to the placebo group (Table 4).

### 3.6. Inflammatory Markers

Inflammatory markers in both groups were within the normal range at the baseline and remained so at the end of the study (Table 4). The change in IL-6 at week 12 was significantly different from placebo according to Wilcoxon Ranks sum tests; however, the significance disappeared after the removal of one single outlier.

### 3.7. Mood Disturbance

**Figure 4:**
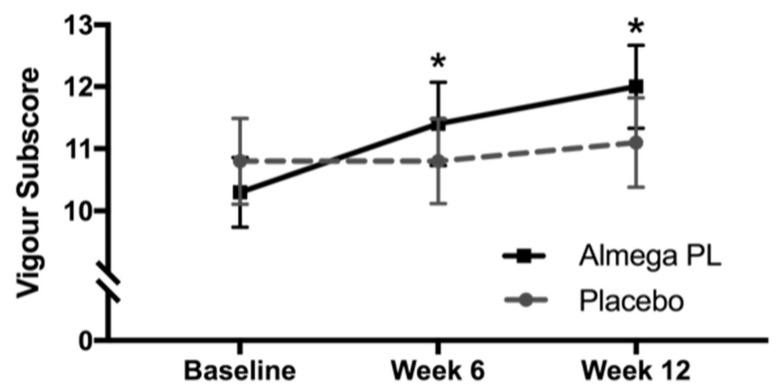
Profile of Mood States (POMS), subscore for Vigour for both the Almega^®^PL group and the placebo group. Values represented as mean ± SEM, * change from baseline significantly different to placebo, *p* < 0.05.

Results indicate that there was a decrease in total mood disturbance in the Almega^®^PL group at both weeks 6 and 12. This indicated that the active treatment may have a positive effect on mood compared to placebo, but this difference was not statistically significant. Looking closer into the individual POMS subscore domains, we found a significant increase in vigor at both weeks 6 and week 12 when compared to placebo (*p* < 0.05) (Figure 4). Tension, Anger, Fatigue, Confusion, Esteem, and Depression scores did not change in either group at any timepoint.

### 3.8. Quality of Life Questionnaires

There were no differences seen in any of the other quality of life measures including general health, sleep, and fatigue. There were no differences in diet or exercise during the study or between groups.

## 4. Discussion

The O3I reflects the dietary intake of this nutrient for up to six months, but typically one-month supplementation is enough to reach the half-maximal concentration [24]. The O3I has been associated with risk for CVD. An O3I of <4% is considered to be associated with a high risk for CVD, with 4–8% having intermediate or moderate risk and >8% having a low risk for CVD [25]. Japan has one of the highest per capita fish consumptions, and therefore its population has a relatively high OI3 (>8%) [26]. Conversely, the USA and Australian population, which eat less fish, have very low O3I levels (<4%) and (<5%) respectively [26] and therefore have a higher risk for CVD [25].

In the current trial, Almega^®^PL treatment increased the baseline O3I from 4.97 ± 0.89 (a level consistent with a Western diet), further away from the high-risk cardiovascular zone to 5.74 ± 0.93% in just 12 weeks of supplementation (Figure 2). The increase in O3I was driven by EPA alone because erythrocyte DHA levels remained constant. This is consistent with the fact that EPA, which is the only LCn-3 PUFA present in Almega^®^PL, does not significantly convert to DHA, but DHA readily converts to EPA in the body [27]. Instead, some EPA is converted to n-3 DPA (Table 2), a fatty acid that is not accounted for in the O3I [28]. Therefore, a source of EPA-only is *a priori* a more difficult way of increasing O3I [29]. However, the EPA present in Almega^®^PL is rich in glycolipids and phospholipids and has higher absorption than other LCn-3 PUFA sources [12]. Both factors may have accounted for the observed O3I increase, which was in line with the response expected from supplements containing both DHA and EPA (±5% of the value predicted by the model for that intake level) [21,22]. Overall, the results show that Almega^®^PL was well absorbed and assimilated, and, despite relatively low supplementation levels (250 mg EPA/day), it provided an effective way of increasing the O3I.

LCn-3 PUFA supplementation improves atherogenic dyslipidemia by decreasing triglycerides and VLDL cholesterol, which along with O3I is associated with the risk for CVD [6]. In the current study, we observed that a daily intake of 1 g of Almega^®^PL can significantly decrease fasting total cholesterol (TC) and VLDL-cholesterol compared to placebo, but we did not observe a decrease in triglycerides. The decreases in blood lipid levels are often a function of baseline levels, such that greater reductions are observed in individuals with higher baseline levels than in healthy normolipidic individuals. For example, when we analyze *post hoc* a subgroup of 76 participants with slightly higher baseline cholesterol levels (>5.5 mmol/L), the reduction in total cholesterol and VLDL was more pronounced than in the whole PP population. These differences were significant after just six weeks of treatment and improved more after 12 weeks. In addition, the subgroup analysis showed a significant decrease compared to placebo on non-HDL-C, which provides a better assessment of CVD risk than TC alone. Perhaps triglyceride levels did not decrease in any of the populations because participant baseline levels (0.99 ± 0.59 PP and 1.08 ± 0.65 for the subgroup) were well below the threshold for borderline hypertriglyceridemia (1.69–2.25 mmol/L) [30]. Regardless, the fact that we observed an impact in total cholesterol and VLDL in the whole PP population is especially relevant because the trial selected for healthy participants and therefore the results represent a broader population.

The decrease in fasting cholesterol was driven by the decrease in the VLDL fraction, which is responsible for transporting the triglycerides assembled in the liver to the muscle and adipose tissue. The liver responds to LCn-3 PUFA supplementation by inhibiting triglyceride production, which contributes to the decrease in VLDL that we observed [10]. Perhaps in alignment with this mechanism, we recorded a significant increase in liver function compared to placebo (i.e., decrease in AST compared to placebo, *p* = 0.0127). In parallel, the decrease in VLDL can also enhance its conversion rate to intermediate-density lipoprotein cholesterol (IDL) and LDL [31], which would further contribute the low VLDL that we observed.

Typically, in DHA containing formulations (i.e., fish oils), VLDL decrease is associated with an increase in LDL [11]. However, the EPA-only based formulations maintain constant LDL throughout the supplementation period, as confirmed in the present study. The capacity to decrease triglyceride and VLDL cholesterol without increasing HDL (and associated cardiovascular risk) appears to be a benefit associated with EPA-only based treatments [7,11], which suggests that physiological differences for EPA and DHA that may impact the cardiovascular outcome. Interestingly, the weight of pharmacological evidence supporting LCn-3 PUFA treatment of CVD is now tipping towards the use of EPA-only, rather than towards DHA and DHA + EPA combinations. Two large clinical studies carried out in Japan (JELIS) [32] and USA (REDUCE-IT) [33] demonstrated that EPA-only can decrease cardiometabolic markers, as well as ischemic events in statin treated patients, by 19% and 25%, respectively, while the most recent large clinical trial evaluating a mixture of DHA + EPA was recently discontinued due to its low likelihood of demonstrating a benefit (STRENGTH) [34].

The above-mentioned pharmaceutical trials [32,33] rely on fish oil to produce ethyl esters from which EPA can be separated and purified from the naturally occurring DHA + EPA mixture. All dietary LCn-3 PUFA sources contain DHA to some extent except for Almega^®^PL, which for the first time, provides a natural source EPA-only. Because the microalga *Nannochloropsis* does not naturally synthetize DHA, further hydrolysis and separation is not required, and EPA can be supplemented in its original polar form (galactolipids and phospholipids). Without the need for extensive purification, Almega^®^PL retains the naturally-occurring active molecules such as phytosterols and chlorophyll, which may have contributed to the non-HDL-cholesterol lowering effect observed in this study [35,36]. The original polar form in microalgae resulted in a better absorption than other LCn-3 PUFA sources [12,37], contributing to the good deposition observed in the current study (i.e., O3I).

In addition, galactolipids are known to inhibit pancreatic lipase activity [38], and in some in vivo studies have shown to reduce appetite (via ileal break) [39], lower fat mass, and promote weight loss [40]. In this study, Almega^®^PL decreased weight by 0.9 kg with respect to the placebo group over 12 weeks (*p* = 0.071). The weight change at week 12 was significantly different from placebo when covariance was analyzed to control for age and baseline BMI. This trend aligned with the decrease in hip circumference of 1.2 cm with respect to the placebo (*p* = 0.029), which encourages further exploration of the potential weight management role of this ingredient. Waist circumference did not significantly decrease, but this may be due to a study population that was dominated by (~60%) female participants. Men tend to accumulate fat in the waist, while women tend to accumulate fat more evenly between the waist and hip [41]. Other potentially-confounding variables, such as exercise and diet intake, remained constant both over time and between treatments and were not considered to contribute to the observed study differences.

Collectively, this study indicates that, compared to the placebo, Almega^®^PL supplementation enhances the omega-3 status in the body, which lowers the total cholesterol and VLDL without increasing HDL cholesterol. In parallel, Almega^®^PL can improve weight management and vigor. While the study extended over only 12 weeks, the participant selection and grouping criteria used suggest that these benefits will be experienced by the general “healthy” adult population. Daily intake of LCn-3 PUFA in the current study (250 mg/day) is consistent with the promotion of cardiovascular health [42], and yet is far from the intake levels (2000–4000 mg/day) recommended to treat CVD [27]. Despite the differences in the recommended intake between healthy and diseased populations, the fact that this study describes some of the same mechanisms associated with EPA treatment (i.e., VLDL lowering without increase in HDL), supports the use of EPA-rich Almega^®^PL to promote cardiovascular health. Futures studies would need to address whether taking more than 1 g/day Almega^®^PL would add extended benefits to these cardiometabolic risk factors. In addition, studies with larger populations and supplementation periods would be necessary to address the benefits of long-term supplementation on both the cardiometabolic risk factors, as well as “hard” CVD endpoints. Despite these limitations, the results are especially relevant because Almega^®^PL is the first natural source of EPA-only that is available over the counter for dietary supplementation. This novel ingredient provides a less processed and more affordable source of this fatty acid that fits the needs of the general population. In conclusion, the Almega^®^PL provides a natural EPA-only option that could increase EPA and O3I, control cholesterol levels, and preventively support cardiovascular health in the general population.

## Figures and Tables

**Table 1:** Baseline participant details.

	Almega^®^PL (*n* = 53)	Placebo (*n* = 51)
Female (*n*)	31	32
Male (*n*)	22	19
Age (years)	53.9 ± 11.0	52.0 ± 12.9
Weight (kg)	77.1 ± 16.0	74.8 ± 14.5
BMI (kg/m^2^)	26.2 ± 4.8	26.4 ± 4.6
Systolic Blood pressure (mmHg)	124.9 ± 16.1	122.5 ± 14.8
Diastolic Blood pressure (mmHg)	81.0 ± 9.1	78.3 ± 8.5
Resting heart rate (BPM)	65.4 ± 9.6	65.7 ± 9.8
Waist circumference (cm)	90.0 ± 13.2	89.4 ± 12.9
Hip Circumference (cm)	104.6 ± 8.9	103.4 ± 8.6

Values represented as mean ± SD, * significantly different from placebo, *p* < 0.05.

**Table 2:** Erythrocyte arachidonic (ARA), eicosapentaenoic (EPA), n-3 docosapentaenoic (DPA) and docosahexaenoic (DHA) acid concentrations as % total fatty acid in blood erythrocytes.

	Almega^®^PL			Placebo		
	Baseline	Week 6	Week 12	Baseline	Week 6	Week 12
ARA	14.83 ± 1.37	15.16 ±1.26	15.38 ± 0.99	14.67 ±1.26	14.76 ± 1.18	15.16 ± 1.14
EPA	0.82 ± 0.37	1.16 ± 0.36 *	1.27 ± 0.36 *	0.82 ± 0.27	0.86 ± 0.00	0.87 ± 0.30
DPA	3.01 ± 0.40	3.45 ± 0.28 *	3.55 ± 0.31 *	3.04 ± 0.58	3.01 ± 0.41	3.08 ± 0.54
DHA	4.18 ± 0.87	4.25 ± 0.82	4.29 ± 0.72	4.46 ± 1.02	4.42 ± 0.89	4.59 ± 1.02

Values represented as mean ± SD, * change from baseline significantly different between groups, *p* < 0.05.

**Table 3:** Total cholesterol, HDL, LDL, VLDL, triglycerides, non-HDL-cholesterol, TC:HDL ratio and triglyceride in plasma (mmol/L).

	**Per-protocol population**					
	**Almega^®^PL (*n* = 53)**			**Placebo (*n* = 51)**		
	**Baseline**	**Week 6**	**Week 12**	**Baseline**	**Week 6**	**Week 12**
Cholesterol	6.47 ± 1.20	6.44 ± 1.16	6.32 ± 1.05 *	6.51 ± 1.48	6.55 ± 1.35	6.54 ± 1.42
HDL	1.87 ± 0.56	1.90 ± 0.56	1.89 ± 0.5	1.77 ± 0.56	1.83 ± 0.53	1.79 ± 0.56
LDL	3.76 ± 0.97	3.80 ± 0.98	3.76 ± 0.83	3.90 ± 1.09	3.93 ± 0.97	3.91 ± 0.97
VLDL	0.91 ± 0.73	0.78 ± 0.49	0.66 ± 0.49 *	0.89 ± 0.59	0.83 ± 0.59	0.89 ± 0.59
Triglycerides	0.99 ± 0.59	1.02 ± 0.66	0.98 ± 0.52	1.04 ± 0.59	1.13 ± 0.54	1.05 ± 0.57
non-HDL-C	4.64 ± 1.20	4.54 ± 1.17	4.41 ± 1.03	4.74 ± 1.39	4.72 ± 1.32	4.77 ± 1.37
TC:HDL Ratio	3.82 ± 1.49	3.71 ± 1.35	3.66 ± 1.29	3.90 ± 1.09	3.81 ± 1.20	3.95 ± 1.44
	**High cholesterol subgroup (>5.5 mmol/L)**					
	**Almega^®^PL (*n* = 40)**			**Placebo (*n* = 36)**		
	**Baseline**	**Week 6**	**Week 12**	**Baseline**	**Week 6**	**Week 12**
Cholesterol	6.94 ± 0.94	6.80 ± 1.05 *	6.65 ± 0.91 *	7.11 ± 1.19	7.09 ± 1.11	7.14 ± 1.18
HDL	1.92 ± 0.59	1.92 ± 0.57	1.91 ± 0.57	1.82 ± 0.60	1.86 ± 0.58	1.82 ± 0.61
LDL	4.09 ± 0.84	4.10 ± 0.91	4.10 ± 0.76	4.33 ± 0.87	4.30 ± 0.81	4.29 ± 0.74
VLDL	1.00 ± 0.77	0.83 ± 0.51	0.73 ± 0.50 *	1.03 ± 0.58	1.01 ± 0.56	1.11 ± 0.60
Triglyceride	1.08 ± 0.65	1.10 ± 0.71	1.03 ± 0.52	1.16 ± 0.62	1.23 ± 0.55	1.15 ± 0.60
non-HDL-C	5.02 ± 1.06	4.89 ± 1.08 *	4.74 ± 0.94 *	5.28 ± 1.12	5.24 ± 1.08	5.31 ± 1.15
TC:HDL ratio	4.03 ± 1.59	3.89 ± 1.39	3.82 ± 1.31	4.22 ± 1.29	4.12 ± 1.18	4.28 ± 1.45

Values represented as mean ± SD, * change from baseline (Δ values) significantly different between groups, *p* < 0.05.

**Table 4:** Inflammatory, general safety markers, and anthropometric results for both the Almega^®^PL group and placebo group.

	Almega^®^PL			Placebo		
	Baseline	Week 6	Week 12	Baseline	Week 6	Week 12
CRP (mg/L)	1.94 ± 2.35	1.47 ± 1.38	2.02 ± 2.88	1.73 ± 2.09	1.64 ± 1.74	2.07 ± 3.15
ALT (U/L)	23.81 ± 14.74	24.56 ± 15.82	22.23 ± 13.62	21.83 ± 12.0	21.09 ± 10.28	23.10 ± 13.82
AST (U/L)	27.85 ± 9.07	29.8 ± 14.10	27.78 ± 9.11 *	26.69 ± 8.27	26.31 ± 8.01	28.20 ± 8.99
HOM (umol/L)	7.72 ± 2.12	7.56 ± 2.12	7.46 ± 1.80	7.03 ± 2.21	7.1 ± 1.93	6.95 ± 1.97
GGT (U/L)	23.21 ± 16.93	22.22 ± 13.58	24.74 ± 16.30	23.86 ± 20.94	23.40 ± 19.33	20.87 ± 14.16
TBIL (umol/L)	9.54 ± 4.34	9.75 ± 5.05	8.73 ± 5.62	8.59 ± 4.22	8.78 ± 3.97	8.16 ± 3.54
CRE (umol/L)	95.55 ± 19.88	106.17 ± 69.14	97.70 ± 18.65	95.06 ± 17.31	96.14 ± 20.56	99.34 ± 16.76
GLU (mmol/L)	5.74 ± 0.59	5.86 ± 0.72	5.89 ± 0.77	5.68 ± 0.69	5.72 ± 0.78	5.72 ± 0.72
IL6 (pg/mL)	8.55 ± 17.04	8.87 ± 16.71	9.43 ± 17.24 *	14.45 ± 26.61	23.76 ± 43.78	22.88 ± 43.69
TNF-α (pg/mL)	7.19 ± 2.96	7.54 ± 4.35	7.86 ± 4.23	7.52 ± 6.08	8.15 ± 6.04	7.65 ± 4.26
d-ROMS (u.carr)	417.5 ± 134.8	404.4 ± 122.0	412.0 ± 104.1	412.3 ± 87.4	402.6 ± 112.9	422.7 ± 96.8
WC (cm)	90.03 ± 13.22	89.76 ± 13.48	89.38 ± 13.19	89.39 ± 12.92	89.43 ± 13.01	90.07 ± 13.21
HC (cm)	104.55 ± 8.89	103.82 ± 8.81	103.86 ± 8.68 *	103.43 ± 8.55	103.40 ± 9.11	104.19 ± 8.98 *
SBP (mmHg)	124.85 ± 16.14	123.17 ± 15.05	121.12 ± 16.40	122.49 ± 14.78	118.98 ± 13.07	120.74 ± 15.52
DBP (mmHg)	81 ± 9.12	79 ± 9.38	78.81 ± 9.79	78.24 ± 8.54	76.22 ± 8.57	78.06 ± 9.11
HR (BPM)	65.36 ± 9.64	67.19 ± 8.46	65.37 ± 9.06	64.69 ± 9.77	66.41 ± 10.43	65.37 ± 9.06
Weight (kg)	77.06 ± 15.98	76.76 ± 15.69	76.79 ± 15.68	74.80 ± 14.49	75.07 ± 14.72	75.22 ± 14.53
BMI (kg/m^2^)	26.20 ± 4.77	26.17 ± 4.59	26.09 ± 4.53	26.35 ± 4.58	26.39 ± 4.60	26.52 ± 4.56

CRP = C-reactive protein, ALT = Alanine transaminase, AST = Aspartate transaminase, HOM = homocysteine, GGT = Gamma-glutamyl transferase, TBIL = total bilirubin, CRE = creatinine, GLU = glucose, IL-6 = interlukin-6, TNF-α = Tumour Necrosis Factor alpha, d-ROMS = The reactive oxygen metabolites, u.carr = Carratelli units, WC = waist circumference, HC = hip circumference, SBP = systolic blood pressure, DBP = diastolic blood pressure, HR = heart rate, BMI = body mass index. Values represented as mean ± SD, * change from baseline (Δ values) significantly different to placebo, *p* < 0.05.
